# Supplementary material for: The caudate nucleus contributes causally to decisions that balance reward and uncertain visual information
Source: eLife. 2020 Jun 22;9:e56694. doi: 10.7554/eLife.56694 (PMC7308093; doi:10.7554/eLife.56694)
Supplement: Supplementary file 1. — Bold: p<0.05. b, Median and p values for microstimulation-induced effects in 39 effective sites, as measured by best DDM fits to the choice and RT data. P values were from Wilcoxon signed rank test. Bold: p<0.05. [file elife-56694-supp1.docx]

**Supplementary File 1**

**a**. Median and p values for microstimulation-induced effects for all 55 sites, as measured by logistic fits to the choice data and linear fits to the RT data. P values were from Wilcoxon signed rank test. Bold: *p*<0.05.

|  | Median | p | Sign. Sites |
| --- | --- | --- | --- |
| Choice: ΔBias (estim) | **0.792** | **0.0144** | 27 |
| Choice: ΔBias (rew x estim) | **0.834** | **0.0035** | 12 |
| Choice: ΔSlope (estim) | **-2.058** | **0.0012** | 14 |
| Choice: ΔSlope (rew x estim) | **-1.818** | **0.0173** | 6 |
|  |  |  |  |
| RT Contra: ΔIntercept (estim) | **-14.788** | **0.0186** | 19 |
| RT Contra: ΔIntercept (rew x estim) | -13.708 | 0.6270 | 10 |
| RT Contra: ΔSlope (estim) | **0.671** | **0.0001** | 11 |
| RT Contra: ΔSlope (rew x estim) | 0.205 | 0.5976 | 4 |
|  |  |  |  |
| RT Ipsi: ΔIntercept (estim) | **-24.251** | **0.0264** | 21 |
| RT Ipsi: ΔIntercept (rew x estim) | **-13.207** | **0.0063** | 11 |
| RT Ipsi: ΔSlope (estim) | **0.448** | **0.0045** | 10 |
| RT Ipsi: ΔSlope (rew x estim) | **0.646** | **0.0000** | 2 |

**b**. Median and p values for microstimulation-induced effects in 39 effective sites, as measured by best DDM fits to the choice and RT data. P values were from Wilcoxon signed rank test. Bold: *p*<0.05.

|  | Median | p |
| --- | --- | --- |
| Δa (estim) | **-0.179** | **<0.0001** |
| Δa (rew x estim) | 0.002 | 0.5819 |
| Δk (estim) | **-0.589** | **0.0003** |
| Δk (rew x estim) | **-0.483** | **0.0062** |
| Δz (estim) | 0.019 | 0.5614 |
| Δz (rew x estim) | 0.026 | 0.3457 |
| Δme (estim) | 0.025 | 0.1156 |
| Δme (rew x estim) | 0.006 | 0.5392 |
| Δt_Contra (estim) | **0.017** | **0.0473** |
| Δt_Contra (rew x estim) | 0.004 | 0.7117 |
| Δt_Ipsi (estim) | **0.016** | **0.0316** |
| Δt_Ipsi (rew x estim) | -0.004 | 0.8034 |
